# Radiological risk assessment and geochemical signatures of calc-alkaline bostonite dikes

**DOI:** 10.1038/s41598-026-45855-w

**Published:** 2026-04-19

**Authors:** Ahmed E. Abdel Gawad, Reham M. Abd El Rahman, Mohamed Y. Hanfi

**Affiliations:** 1 Nuclear Materials Authority, El-Maadi, P.O. Box 530, Cairo, Egypt; 2 Ural Federal University, Ekaterinburg, 620002 Russia; 3 Department of Physics, Dogus University, Dudullu-Ümraniye, 34775 Istanbul, Türkiye

**Keywords:** Bostonite, Radiological hazards, Geochemical signatures, Gamma-ray spectrometry, Multivariate statistical analysis, Environmental sciences, Natural hazards

## Abstract

The bostonite dikes in the studied area of Egypt are one of the most radioactive felsic intrusions found in the South Eastern Desert due to their elevated levels of natural occurring radionuclides and the radiological hazards associated with them. This study presents an integration of geochemical signatures and gamma-ray spectrometry data to quantify the activity concentrations of ^238^U, ^232^Th, and ^40^K found in bostonite samples. Geochemical characterization was performed using ARL X-ray fluorescence, while radioactivity was measured using a calibrated sodium iodide (NaI(Tl)) gamma-ray spectrometer. The geochemical signatures of the investigated bostonite dikes from El Sela-Qash Amir area reveal that the analyzed samples fall in trachyte field, calc-alkaline affinity. They are distinguished by remarkable higher concentrations in large-ion lithophile elements (LILEs) and high field strength elements (HFSEs). Gamma-ray investigations have revealed significant and variable levels (Mean ± standard deviation) of ^238^U (150 ± 47 Bq kg^− 1^), ^232^Th (103 ± 17 Bq kg^− 1^), and ^40^K (1379 ± 182 Bq kg^− 1^) compared to average global crustal values. The statistical results of multivariate analysis (including Pearson’s correlation coefficient, hierarchical cluster analysis, and principal component analysis) indicated that ^238^U and ^40^K were the most significant contributors to the variation observed in radiological hazard parameters across the bostonite. Based on the radiological hazard parameters calculated, samples of bostonite near their highest acceptable limits and ELCR calculations indicate that long-term exposure may have a detrimental health impact. These results demonstrate that bostonite disturbances have significant radiological importance and warrant future monitoring and regulation due to the health effects of the bostonite rocks at studied area.

## Introduction

 The uneven distribution of natural radioactive isotopes (^238^U, ^232^Th and ^40^K) within igneous rocks is determined by many factors including the mineralogical composition and geochemical evolution of each rock. Within felsic intrusions (specifically alkaline and peralkaline igneous rock systems), a number of accessory minerals (uraninite, coffinite, pitchblende, brannerite, uranophane, autunite, thorianite, thorite, brockite, uranothorite, phosphothorite, monazite and zircon) typically enrich these isotopes^1–7^. Natural Radiation is the largest source of radiation exposure to much of the general population. It is important to identify contaminated areas with elevated levels of natural background radiation so as to assess the overall environmental risk and set a standard for the remediation of these sites. In order to do this, measurement of naturally occurring radioactivity from the environment in each local area should be made^8–10^.

Uranium is mobile at shallow depths compared to thorium, which remains static. The oxidation process of uranium creates a water-soluble version of the uranium, allowing for diaspora away from its rock formation through the process of leaching. Thus, water-soluble uranium is capable of creating lateral migratory patterns through solutions and depositing into sediments after migrating away from a source. Conversely, due to lower solubility, thorium and potassium showing higher concentration coincided with altered zone^6,7^. Felsic rock types contain a high level of both thorium and uranium, particularly granites and their pegmatite. The alkalinity and/or acidity of the geochemical properties of these rocks plays a role in both thorium and uranium abundance and the alkaline nature of granite leads to an increase in potassium content. By examining the natural levels of thorium and uranium in both the rocks and soils, we can establish a basis for assessing the radiological effects of thorium and uranium on humans in relation to property development, safety concerns associated with building residential properties on soil and rock^11,12^.

Dimensional stones are identified as gifts of nature as hard rocks that have remarkable versatile applications such as ornamental, building, sculptural and decorative stones as well as aggregate stones. They including bostonite, trachyte, rhyolite, basalt, granite, pegmatite, limestone, sandstone and marble, and are used in the construction industry, like arrowhead, as gemstones particularly in decorating garden and coating stones^13–15^. Dimensional stones are also utilized in stone huts, building defensive walls, fairies, knives, ceramic, cutting tools, road aggregates, bridges, monuments and arches^16^. Rhyolite and bostanite are well recognized for their propensity to have higher levels of natural radionuclides, which could be attributed to the presence of accessory minerals such as uranophane, uranothorite, thorite and monazite as well as zircon^17,18^. These minerals exhibit a remarkable tendency to concentrate thorium and uranium. Consequently, the distribution of radionuclides and their associated radiological hazards in bostonite is of substantial environmental and health concern^7,19^. In Egypt, bostonite reveal remarkable valuable strategic and economic importance as a good resources of rare metals such as Nb, Ta, Zr, Sn, F, Y, REEs, U and Th, which are widely distributed particularly in granites, pegmatites, mylonite, rhyolite flow tuffs, lamprophyre dikes and jasper veins^20–26^.

While numerous of the existing studies on natural radioactivity within granitic and associated rock types have been done in the field, much work remains to be done to characterize the radiological and geochemical characteristics of the bostonite dikes located in the El Sela Shear Zone. The bostonite located in El Sela-Qash Amir contain rare-metals and uranium-rich minerals. Therefore, the objective of this study is to evaluate the radiological risks associated with exposure to natural radionuclides in bostonite-bearing mineralization using a gamma-ray spectrometer utilizing the NaI (Tl) method for bulk analysis and then conducting multivariate statistical analysis (using Pearson Correlation, PCA & HCA) to determine how closely linked the accessory radioactive minerals are to the activity concentrations and hazard indices measured. The study identifies hazards raised from excessive exposure to radioactive materials by estimating various parameters related to radiological risks, providing novel insights into the geochemical behaviour of the analyzed major and trace elements, radioactivity levels, and potential health and environmental implications of using bostonite in construction materials.

## Geologic setting

**Fig. 1:**
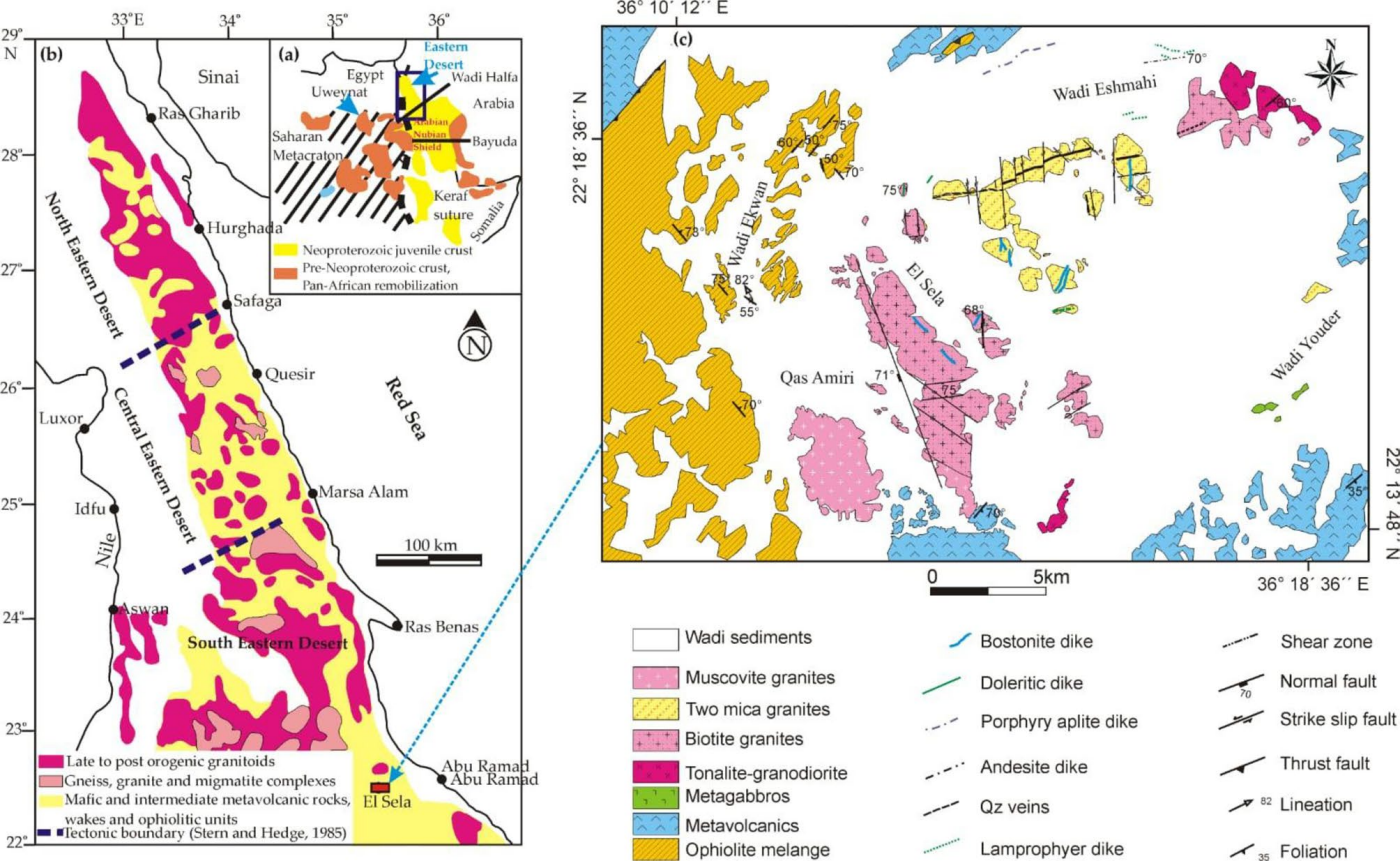
(**a**) Geologic map shows the Arabian Nubian Shield (ANS); (**b**) Geologic map shows the Neoproterozoic basement complex in the ED of Egypt^27^; (**c**) Geologic map of El Sela-Qash Amir region, SED of Egypt after^29,32^. The map is drawn by Coreldraw 12 software for free trial (https://www.coreldraw.com/en/).

The Eastern Desert (ED) of Egypt is well identified as a part of the so called the Arabian–Nubian Shield (ANS). The ANS comprise Precambrian basement complexes that are widely exposed along the two flanks of the Red Sea in the Western Arabian and the northeaster Africa. It comprises Egypt, Sudan, Ethiopia, Eritrea, Somalia, Saudi Arabia, Jordan, Oman, and Yemen (Fig. 1a,b). El Sela-Qash Amir region is considered as one of the most important location in the South Eastern Desert (SED) of Egypt. It is selected as a case study of the (ANS)^27^ (Fig. 1a,b).

**Fig. 2:**
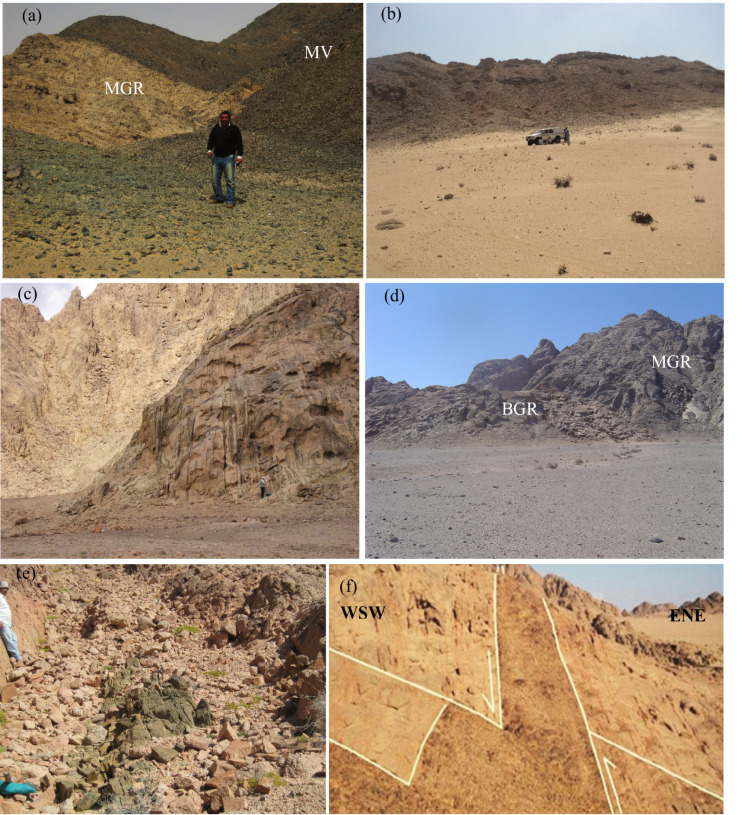
(**a**) Intrusive contact between the muscovite granite (MGR) and metavolcanics (MV), (**b**) ENE-shear zone affected the two-mica granite, (**c**) Mn-minerals filling micro-fractures in the muscovite granite, (**d**) Sharp intrusive contact between the biotite granite (BGR) and muscovite granite (MGR), (**e**) dolerite dikes dissected the two-mica granite along ENE-trend; (**f**) Bostonite dike invades two-mica granite, and affected by the N–S strike slip fault crosscut ENE–WSW fault causing distinct left movement.

It is enriched in radioactive bearing two-mica granite as well as manganese and tungsten ore deposits that attract many researchers^28–30^. From field investigation, El Sela-Qash Amir region consists of the ophiolitic mélange, metavolcanics, older granitoids, and granitic rocks varying from the biotite granite, two-mica granite and muscovite granite that were dissected by microgranite, dolerite and bostonite dike swarms, as well as quartz and jasper veins (Figs. 1c and 2a–f).

Sol Hamed ophiolitic mélange rocks occur in the western part of the El Sela-Qash Amir area. These are represented by serpentinite, metagabbro and basalt. Serpentinite rocks distinguished by their fine-grain, massive, pale gray to grayish violet colors form conspicuous mountainous ridges with remarkable steep slopes, while metagabbro occurs as medium-grain, small masses, highly altered, and occasionally deformed and highly foliated. The matrix of mélange is biotitic muscovite schist, actinolite schist and quartz–feldspathic schist. Serpentinite overlain the metagabbro throughout a thrust fault as mentioned in Fig. 1c.

Metavolcanics occur as fine-grain, buff, pale green to grayish green color, form a thick sequence of stratified lava flows interbanded with their pyroclastics in the northern and southern parts of the investigated area (Fig. 1c). They consist of metarhyolite, metadacite, meta-andesite and, to a lesser extent, of metabasalt. These volcanic rocks interbanded with metatuffs and agglomerate. Metatuffs comprising ash, lithic and lapilli metatuffs crop out as a thin belt in the northwestern part of the study area. Younger granites intruded the metavolcanics with remarkable sharp intrusive contacts (Fig. 2a).

Tonalite-granodiorite occur as small scattered masses in northern and southern parts of the prospected area. These rocks are distinguished by low-lying relief and scattered blocky appetences. They occur as medium-to coarse-grained, greenish grey to whitish grey color and composed of plagioclase, hornblend and biotite. They are highly fractured, jointed, weathered. They intruded the ophiolitic mélange and metavolcanics.

Biotite granite is well exposed in the investigated area have NNW-trend. It is pinkish to buff red colors, medium- to coarse-grain, massive, leucocratic, and exhibiting low-lying to moderate relief. This granite masses is highly jointed, fractured and showing cavernous and bouldery appetences. It consists mainly of quartz, K-feldspar, plagioclase, biotite and minor muscovite. It contains xenoliths of black color of metavolcanics.

The two-mica granite is the main host rock type in the investigated El Sela-Qash Amir region. It is characterized by medium- to coarse-grained, moderate- to high-relief masses, pink to reddish buff colors. It is highly weathered, fractured, jointed and strongly exfoliated. This granite is predominated affected by ENE-shear zone (Fig. 2b), as well as NW and N–S strike-slip faults. It is composed mainly of quartz, K-feldspar, plagioclase, muscovite and biotite.

Muscovite granite occurs in the southwestern part at Qash Amir. It manifest as oval and isolated hill, highly foliated, medium to coarse-grain, buff to whitish red colors and showing cavernous features due to weathering processes. It consists of quartz, K-feldspar, plagioclase, muscovite and biotite. At the peripheries of Qash Amir plutons, Mn-dendritic shape filling microfractures along the E–W and N–S trends (Fig. 2c). This granitic rock is dissected by quartz veins bearing tungsten minerals, violet to green color of fluorite, Mn-minerals, pyrite, galena, silver and gold^31^. The biotite granite was intruded by the two-mica granite and muscovite with intrusive sharp contact (Fig. 2d).

Microgranite dikes manifest as fine-grained, buff to reddish, and gray colors as a result of clay alteration. They invaded the monzogranite along ENE shear zone (Fig. 1c). They were varied in thickness from 2 to 25 m. They are composed mainly of quartz, K-feldspar, plagioclase, muscovite and biotite. These dikes were subjected to an extensive hydrothermal fluids, and became completely to slightly altered into clay minerals along the main shear zone (Fig. 1c).

Dolerite dikes are fine-grained, grayish green to gray colors, highly weathered, fractured, jointed and sheared (Fig. 2e). They range in their thickness from 1.5 to 12 m. They were have dissected the monzogranite along the ENE and NNW main shear zones. They are composed mainly of plagioclase, olivine and pyroxene as well as visible uranophane, autunite and pyrite megacrysts.

Bostonite dikes manifest as fine-grained, burned brown to reddish colors, and range from 1 to 5 m thick. They were dissected the granitic rocks along N–S structural trend (Fig. 2f). They are consisted of alkali feldspar phenocrysts, amphiboles, with lower content of quartz that embedded in fine-grained groundmass.

Quartz and jasper veins are distinguished by their white milk to bright white, black to gray and reddish brown colors. They were invaded the monzogranite along the main shear zone have ENE-structural trend. They are varied in their width from 1.5 to 6 m thick. These veins are highly fractured, jointed and affeted by ENE-main shear zone, and are enriched in visible dendritic Mn-minerals, megacrystals of cubic pyrite and visible uranophane.

El Sela-Qash Amir region is extensively affected by the two perpendicular fault systems have ENE and NNW structural trends. They could be played a crucial role as a remarkable good pathway for the acidic and/or alkaline hydrothermal alteration (silicification, argillization, fluoritization and hematitization). It controls multi-injections that is enriched in uranium mineralization, that have been deposited along the main shear zones.

## Materials and methods

### Sampling, preparation and analysis

The Nuclear Materials Authority (NMA) in Cairo received 50 bostonite rock samples (approximately 250 g each) taken from the El Sela-Qash Amir region for thorough analysis through radiometric methods. When the samples were received at the NMA, they were first disaggregated (crushed and sifted) in the Radiation Protection Laboratory before being chemically labelled and packaged in transparent polyethylene bags to allow for tracking them through the different phases of analysis at the NMA.

A gamma spectrometric analysis using a calibrated NaI(Tl) detector was performed on samples dried for 72 h at 100 °C, sieved (2 mm), and placed in 250 mL containers and sealed for four weeks to ensure secular equilibrium between radon (^222^Rn) and its decay products in both the uranium and thorium decay series, which are necessary for the accurate estimate of the activity of ^238^U and ^232^Th based on their corresponding gamma-emitting daughters. The system consisted of a cylindrical crystal (7.6 × 7.6 cm) designed for environmental radioactivity measurements. The detector was housed in a cylindrical lead shielding assembly (0.157 m diameter, 0.205 m height, 0.037 m thick) to minimize background radiation and improve the precision of low-level measurements. All data acquisition was done using an amplifier connected to the multichannel analyzer, and all components were controlled via SPTR-ATC (AT-1315) software. After collecting the spectrums, the isotopes count rates associated with the specific photo peaks for each radionuclide were identified and used to calculate the FWHM of the detector and to quantify the activities. Uranium was determined using the gamma lines from the two short-lived daughters of uranium, ^214^Pb (351.9 keV) and ^214^Bi (609.2 keV). Thorium activity was calculated from many of the defined peaks; such as 911.2 keV (26%) and 969.0 keV (16%) for ^228^Ac, 583.2 keV (30.5%) and 2614.5 keV (35.8%) for ^208^Tl, and 238.6 keV (43.6%) for ^212^Pb. All of these peaks were exceptionally useful in determining the activity and concentration of all of the ^232^Th series isotopes in secular equilibrium. The activity concentration of ^40^K was determined through the employed 1460 keV photopeak (10.66%)^33,34^. IAEA-certified reference materials RGU-1, RGTh-1 and RGK-1 were used for calibration of the detectors. For detector efficiency, a standard reference geometry that exactly matched the Marinelli beaker sample configurations was also used to eliminate any geometrical discrepancies between the detector calibration standards and bulk sample measurements^35–37^. The Minimum Detectable Activity (MDA) value for each of the three radionuclides (^238^U, ^232^Th, ^40^K) is then calculated for each of the samples. This process involves taking into account the weight of the sample in order to compute the MDA based upon the sample weight^38^.

1 $$\:MDA\:=\:\frac{2.7+\:4.65\:\surd\:B\:}{\:M\epsilon\:{I}_{\gamma\:}t},\:$$

The mass of the sample (M); background count under the peak (B); efficiency of absolute (ε); gamma-ray intensity (Iγ); counting time in seconds (t). The MDA values for all samples recorded for up to 20,000 s were: ^238^U − 2 Bq kg^− 1^; ^232^Th − 4 Bq kg^− 1^ and ^40^K − 12 Bq kg^− 1^. Total uncertainty in all radiation levels is calculated from the deviation equation considering both normal and stochastic errors (misspecifications). Radioactivity reading during efficiency calibration represent an overall systematic uncertainty of 0.5 to 2% and random variations of up to 5%^39^. Relative to the respective concentration (Bq kg^− 1^) of each sample, the following conversions have been applied: respectively for 313 Bq kg^− 1^, 12.35 Bq kg^− 1^, and 4.06 Bq kg^− 1^, 1% K-40, 1 ppm U-238 and 1 ppm Th-232^40^. The following derived metrics are used for estimating background radiation levels: radium equivalent activity (Ra_eq_), indoor and outdoor hazard indices (H_in_ and H_ex_) absorbed dose rate (D_air_, nGy/h), annual effective dose (AED, mSv y^− 1^) and excess lifetime cancer risk factor (ELCR×10^− 3^).

Radium equivalent activity (Ra_eq_) is a cumulative value for assessing the potential radiological harm from natural radioactive materials. The radium equivalent activity combines the activities of ^238^U, ^232^Th, and ^40^K into a simple common factor, which allows for easier assessment of potential health impacts of exposure to gamma radiation. An acceptable maximum radium equivalent activity is defined as 370 Bq kg^− 1^ as per international safety recommendations; if the maximum annual effective dose is less than 1 mSv, the material will present no radiological risk to human health^41^.

2 $${\rm{R}}{{\rm{a}}_{{\rm{eq}}}}({\rm{Bq k}}{{\rm{g}}^{ - {\rm{1}}}}){\rm{ }} = {\rm{ }}{{\rm{A}}_{\rm{U}}} + {\rm{ 1}}.{\rm{43 }}{{\rm{A}}_{{\rm{Th}}}} + {\rm{ }}0.0{\rm{77 }}{{\rm{A}}_{\rm{K}}}$$

External and internal radiation hazard indices (H_ex_ and H_in_) are used to determine the impact of radiation on human health. The danger indices are calculated using Eqs. (3) and (4)^42,43^:

3 $${{\rm{H}}_{{\rm{ex}}}} = \:\frac{{{{\rm{A}}_{\left( {238{\rm{U}}} \right)}}}}{{370}} + \:\frac{{{{\rm{A}}_{\left( {232{\rm{Th}}} \right)}}}}{{259}}{\rm{ + + }}\:\frac{{{{\rm{A}}_{\left( {40{\rm{K}}} \right)}}}}{{4810}} \le {\rm{1}}$$

4 $${{\rm{H}}_{{\rm{in}}}} = \:\frac{{{{\rm{A}}_{\left( {238{\rm{U}}} \right)}}}}{{185}} + \:\frac{{{{\rm{A}}_{\left( {232{\rm{Th}}} \right)}}}}{{259}}{\rm{ + + }}\:\frac{{{{\rm{A}}_{\left( {40{\rm{K}}} \right)}}}}{{4810}} \le 1$$

H_ex_ and H_in_ should not exceed 1.0 ^44,45^.

The gamma radiation received in the region of 1 m was assessed to determine the absorbed dose rate (D_air_). The annual effective dose for both AED_out_ and AED_in_ was estimated, based on the occupancy factor of 0.2 for AED_out_ and 0.8 for AED_in_. The detailed description of the various methodologies to estimate the annual effective dose are provided in the following sections^46,47^.

5 $${{\rm{D}}_{{\rm{air}}}}\left( {{\rm{nGy }}{{\rm{h}}^{ - {\rm{1}}}}} \right){\rm{ }} = {\rm{ }}0.{\rm{43}}0{\rm{ }}{{\rm{A}}_{\rm{U}}} + {\rm{ }}0.{\rm{666 }}{{\rm{A}}_{{\rm{Th}}}} + {\rm{ }}0.0{\rm{42 }}{{\rm{A}}_{\rm{K}}}$$

6 $$\:AED\left(\frac{mSv}{y}\right)=\:\sum\:\left({D}_{air}\:\left(\frac{nGy}{h}\right)\times\:0.7\:\left(\frac{Sv}{Gy}\right)\times\:occupancy\:factor\right)\times\:8760h\:\times\:{10}^{-6}\:\:$$

The AGDE, which is an alternative form of measuring the hazard from radiation, is also involved in the yearly determination of the Dair from the whole body. For ^238^U, ^232^Th and ^40^K, the AGDE is calculated based on the amount of gamma radiation emitted (Eq. (7)^48^).

7 $${\rm{AGDE }}\left( {\mu {\rm{Sv }}{{\rm{y}}^{ - {\rm{1}}}}} \right){\rm{ }} = {\rm{ 3}}.0{\rm{9}}{{\rm{A}}_{\left( {238{\rm{U}}} \right)}}~ + {\rm{ 4}}.{\rm{18}}A{{\rm{A}}_{\left( {232{\rm{Th}}} \right)}}~ + {\rm{ }}0.{\rm{314}}{{\rm{A}}_{\left( {40{\rm{K}}} \right)}}$$

The likelihood of developing cancer throughout the life span (≈ 70 years) caused by exposure to gamma radiation is referred to as the Excess Lifetime Cancer Risk (ELCR), which can be calculated using Eq. (8)^49^, where cancer risk factor (RF = 0.05 Sv^− 1^).

8 $$\:\mathrm{E}\mathrm{L}\mathrm{C}\mathrm{R}\left(\mathrm{m}\mathrm{S}\mathrm{v}/\mathrm{y}\right)=\:\mathrm{A}\mathrm{E}\mathrm{D}\mathrm{o}\mathrm{u}\mathrm{t}\:\mathrm{x}\:\mathrm{D}\mathrm{L}\:\mathrm{x}\:\mathrm{R}\mathrm{F}$$

### Geochemical analysis (XRF)

In this study, ten bostonite samples were selected, then crushed and powdered using agate mortar yielding a powdered grain size of 200 mesh in order to avoid contamination. The major oxides and trace elements were have analyzed utilizing X-ray fluorescence (ARL 9800 f). The ARL X-ray spectrometer at the Central Laboratories of St. Petersburg State University, Russian Federation. The XRF analyses of the bostonite powder samples were have prepared utilizing Mowiol II polyvinyl alcohol, and then fused with tetraborate pellets. The detection limits of the analyzed samples ranges from 1 to 4 mg kg^− 1^ for the trace elements and 0.01% for the major oxides.

## Results and discussion

### Geochemical signatures of Bostonite dikes

Whole-rock chemical analyses of the analyzed major oxides and trace elements concentration of the studied investigated bostonite dikes are listed in Table 1. The geochemical behavior of major oxides of the investigated samples reveal that SiO_2_ ranges from 64.12 to 65.85 wt%, Al_2_O_3_ ranges from 14.44 to 16.17 wt%, the Na_2_O content ranges from 4.43 to 5.79 wt%, and the K_2_O content from 5.47 to 6.71 wt%, and CaO ranges from 2.51 to 3.22 wt%. Ferromagnesian oxides particularly Fe_2_O_3_ reveal high concentrations and reached up 5.52 wt%, whereas MgO and MnO reveal low concentrations, and reached up 0.54 and 0.24 wt%, respectively. The analyzed bostonite samples show that trace elements showing a remarkable enrichment in large-ion lithophile elements (LILEs) particularly Ba, Rb and Sr with averages 339, 198 and 116 mg kg^− 1^, respectively. The high field strength elements (HFSEs) including Zr, Nb and Y show significant higher concentrations with averages 1403, 296 and 110 mg kg^− 1^, respectively.

**Fig. 3:**
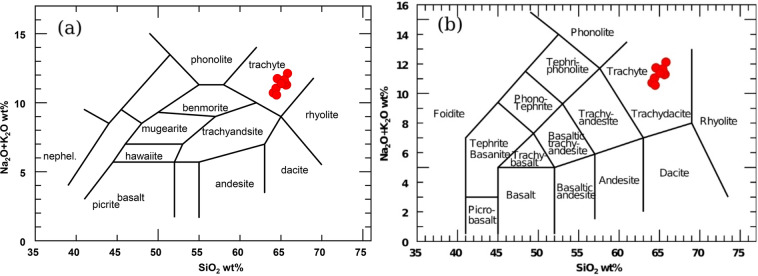
The total alkalis (Na_2_O+K_2_O) versus silica (SiO_2_) diagrams according to (**a**)^50^ and (**b**)^51^.

Many geochemical classifications proposed diagrams have been done for igneous rocks identification utilized various geochemical parameters. The total alkalis (Na_2_O+K_2_O) versus silica (SiO_2_) (TAS) diagrams^50,51^ show that the analyzed samples are plotted in plotted in the trachyte field (Fig. 3a,b).

**Fig. 4:**
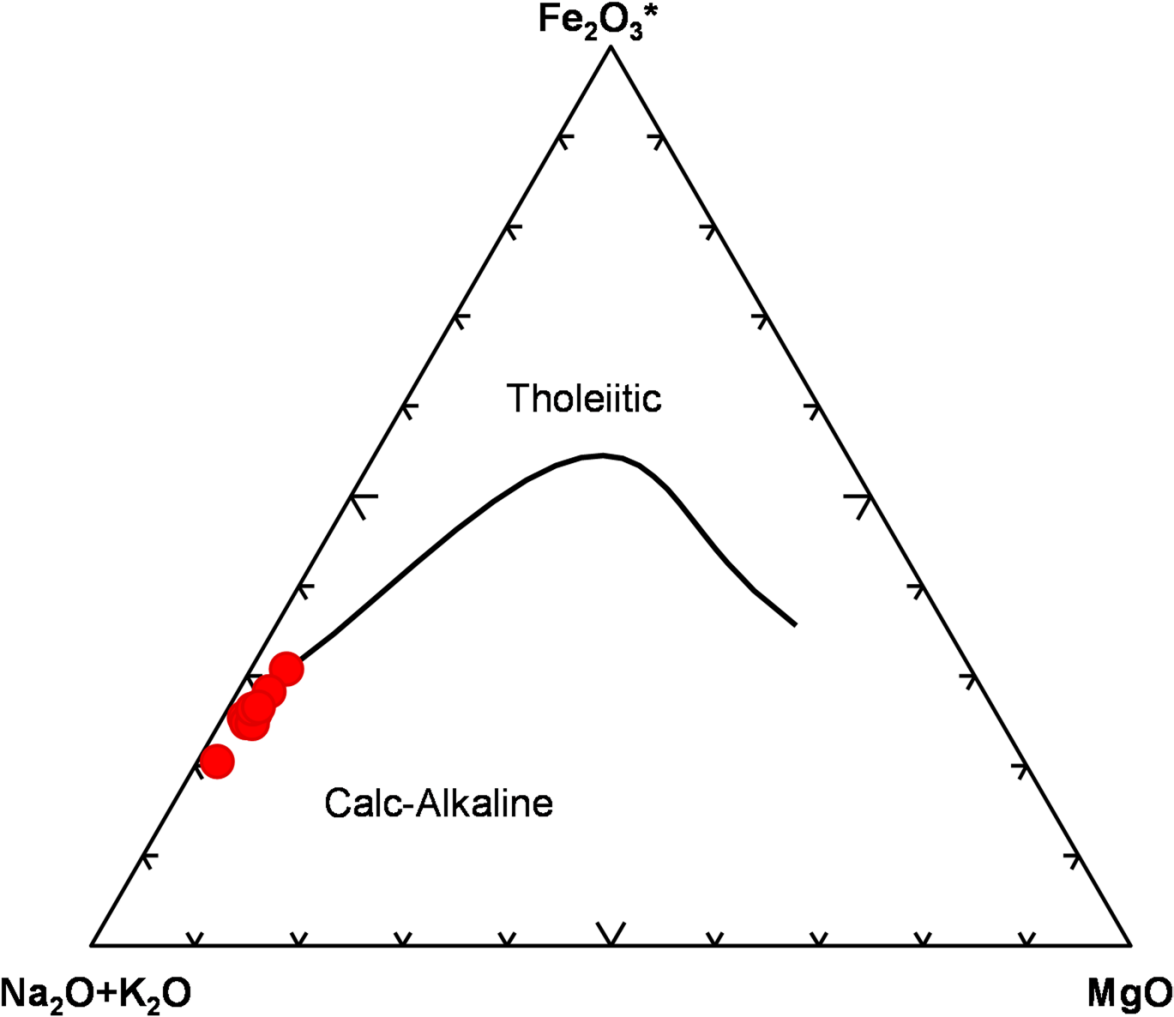
(Na_2_O+K_2_O)-Fe_2_O_3_-MgO diagram (AFM) according to^52^ showing geochemical affinities of the analyzed bostonite samples.

AFM diagram^52^, used to discriminate the tholetic from the calc-alkaline domain. All the analyzed bostonite samples are placed in the calc-alkaline field (Fig. 4).

**Table 1:** Representative chemical analyses of the investigated bostonite samples at El Sela-Qash Amir region, SED of Egypt.

	S47	S30	S21	S3	S44	S39	S8	S49	S35	S13
Major elements oxide (wt%)										
SiO_2_	64.12	65.75	64.44	65.66	64.51	65.85	64.62	65.55	65.46	65.1
Al_2_O_3_	15.53	14.98	16.17	15.12	16.16	15.04	15.17	14.78	14.44	14.47
TiO_2_	0.35	0.18	0.27	0.26	0.22	0.11	0.33	0.34	0.25	0.24
Fe_2_O_3_	5.52	4.35	4.43	4.24	4.79	3.52	4.7	4.32	4.74	4.71
MnO	0.24	0.11	0.18	0.12	0.2	0.08	0.21	0.14	0.17	0.19
MgO	0.6	0.36	0.48	0.44	0.50	0.34	0.49	0.54	0.43	0.50
CaO	2.69	2.76	2.74	2.65	2.83	2.72	2.51	2.67	2.62	3.22
Na_2_O	4.43	5.79	5.14	5.75	5.06	5.38	5.64	5.05	5.10	5.49
K_2_O	6.26	5.48	5.87	5.50	5.47	6.71	6.07	6.38	6.53	5.86
P_2_O_5_	0.06	0.05	0.06	0.05	0.07	0.05	0.05	0.05	0.05	0.06
LOI	0.2	0.19	0.22	0.21	0.19	0.2	0.21	0.18	0.21	0.16
Total	100	100	100	100	100	100	100	100	100	100
Trace elements (mg kg^− 1^)										
Ba	342	331	336	293	340	290	386	340	338	391
Co	6.35	7.05	5.17	6.69	3.63	4.98	4.69	5.57	8.28	6.20
Ga	98.35	53.10	66.72	67.17	59.71	52.60	67.17	63.76	68.92	69.52
Nb	471	154	312	329	297	155	329	297	307	312
Cu	17.4	11.52	17.96	23.53	19.84	18.05	16.45	20.14	15.45	15.41
Pb	15.73	13.95	27.34	14.89	26.13	22.81	18.91	10.55	16.43	19.38
Zn	378	119	247	229	266	113	236	260	245	257
Ni	385	181	286	242	302	148	289	298	295	292
Rb	225	157	206	194	218	142	194	215	212	213
Sr	148	144	107	110	104	99.8	119	115	108	101
Th	23.78	25.39	24.05	23.25	24.67	24.54	19.33	22.66	25.44	18.11
U	15.88	10.25	12.92	14.79	14.08	17.21	9.59	14.05	14.93	9.35
Y	148	143	115	111	97.9	100	106	95.7	96.5	91.4
Zr	2224	716	1470	1300	1667	743	1400	1562	1501	1449
Th/U	1.50	2.48	1.86	1.57	1.75	1.43	2.02	1.61	1.70	1.94

**Fig. 5:**
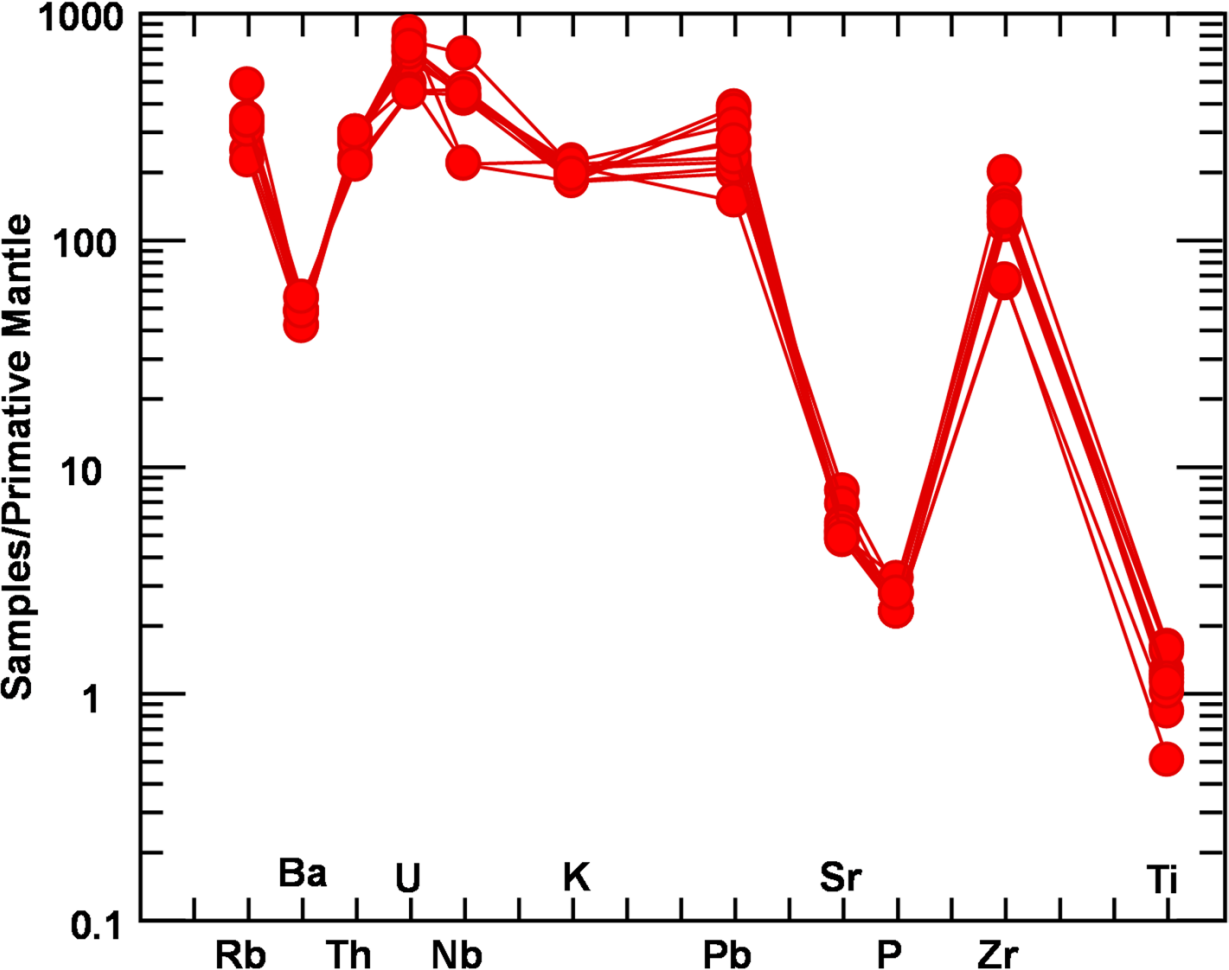
Multi-elements pattern of the analyzed bostonite samples, and the primitive mantle Normalized values (mg kg^−1^) according to^53^.

The normalized multi-elements pattern to the primitive mantle^53^ could be a good and useful indicators to give a general view of the main source and tectonic affinities of the analyzed bostonite samples. Figure 5 shows remarkable enrichment especially in large ion lithophile elements (LILE) especially Rb, Ba and Pb), with those of high field strength elements (HFSE) paticularly Nb, and Zr. The bostonite samples display an obvious marked troughs (negative anomalies) particularly in Ba, P and Ti with respect to their neighbors. The negative Ti anomaly could be associated with the fractionation of titano-magnetite, while the depletion of Ba and Sr reflecting the presence of apatite and feldspars in the fractionating assemblages and/or melt residues.

**Fig. 6:**
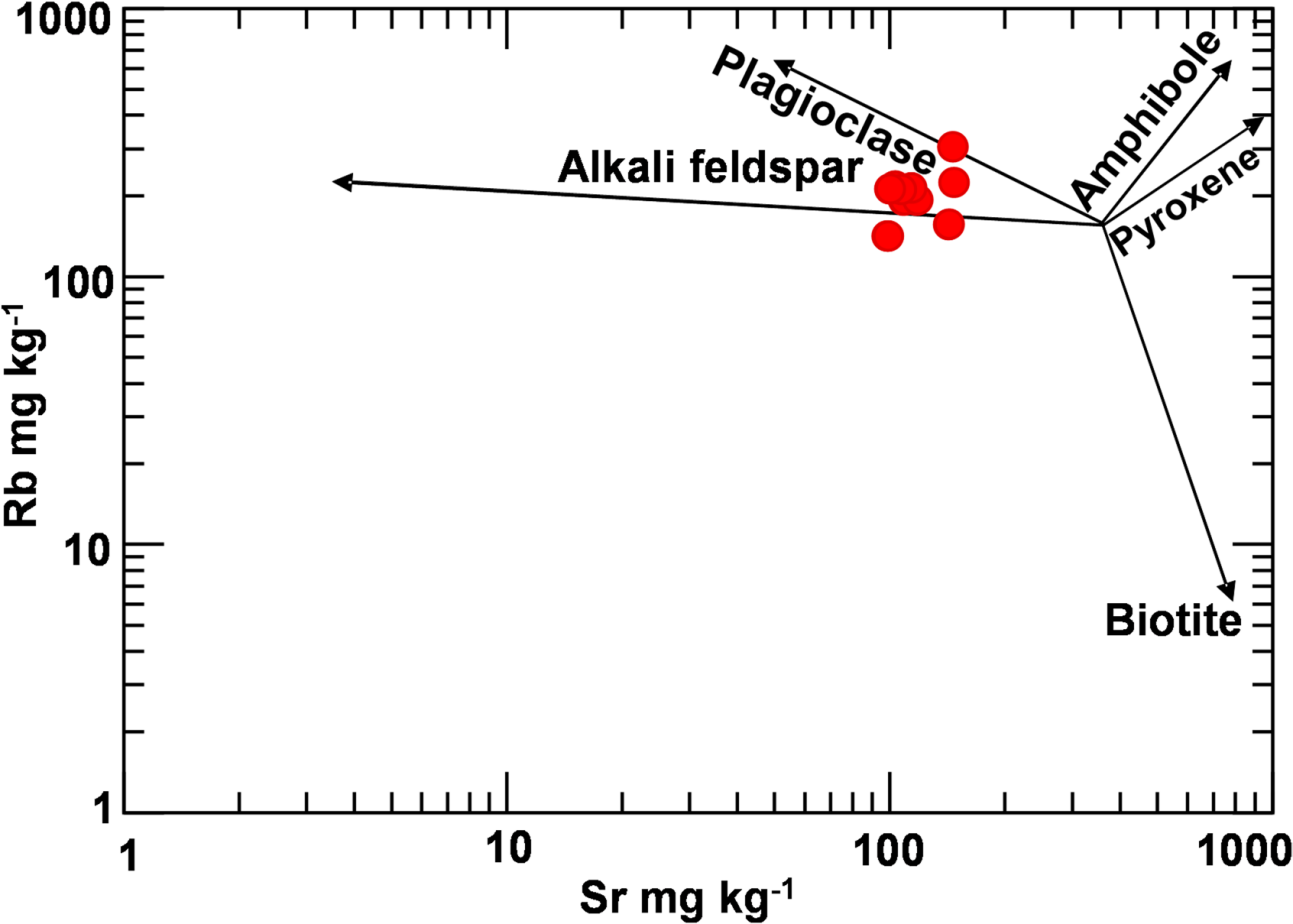
Binary Rb versus Sr diagram showing the petrogenetic aspects of the analyzed bostonite samples.

Figure 6 binary diagram of Rb versus Sr of the analyzed samples showing the role of alkali feldspar and plagioclase fractionation in the investigated bostonite dikes.

**Fig. 7:**
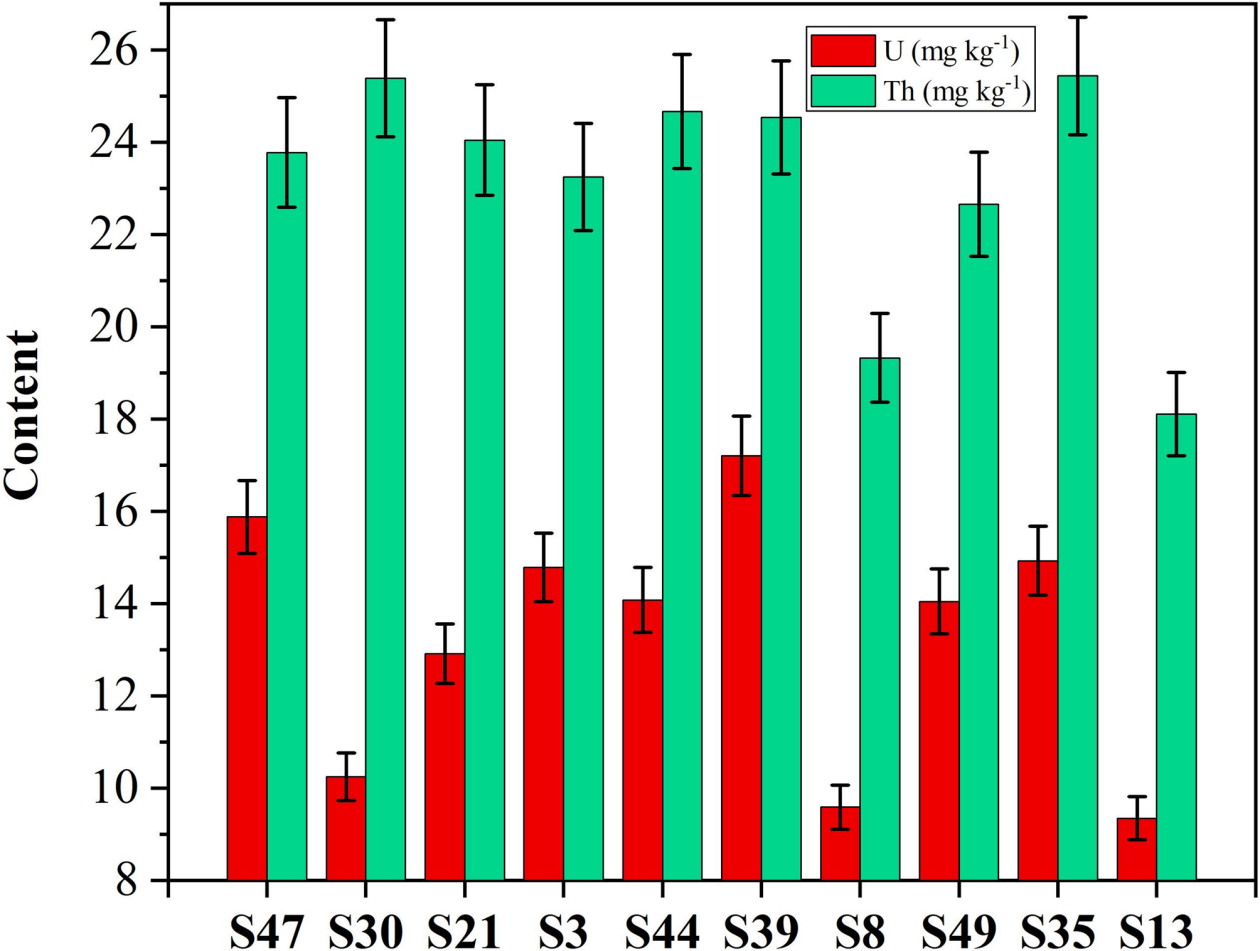
Histogram shows the distribution of U and Th in the investigated bostonite samples.

Figure 7 presents the distribution of U and Th in the analyzed bostonite samples. The geochemical concentrations of is well presented in the histogram which reveal that Th ranges from 18.11 to 25.44 mg kg^− 1^, and U ranges from 9.35 to 17.21 mg kg^− 1^ (Table 1). The Th/U ratios range from 1.43 to 2.48, which seem to considered lower than average of the upper continental crust (3.8^54^). This could be attributed to the uranium and thorium enriched together magmatic evolution.

### Radioactive content in bostonite samples

**Fig. 8:**
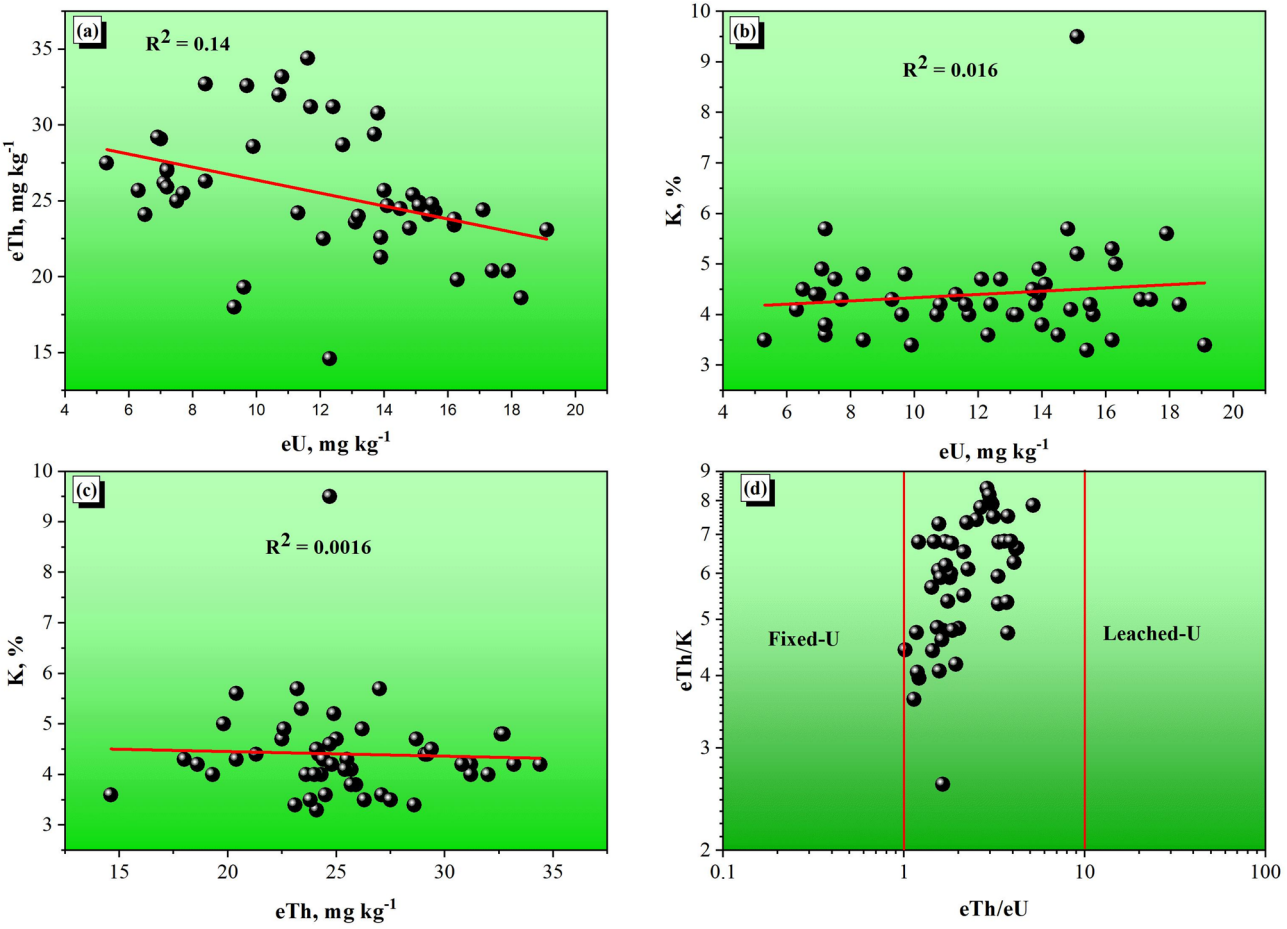
Radiometric analysis in 50 samples of bostonite in the studied region: correlations between (**a**) eTh and eU, (**b**) eU and K, %, (**c**) eTh and K,%, (**d**) eTh/K and eTh/eU.

Figure 8 shows that relationships exist between the eU, eTh and K of the bostonite samples included in this study, which gives insight into both the radioactivity and geochemical behaviour of the analyzed samples. From the eTh–eU diagram in Fig. 8a, very weak correlation (R^2^ = 0.14), between thorium and uranium shows that there are no coherent changes in either of these elements, which indicates that almost all of the observed decoupling of uranium and thorium has occurred after the formation of the bostonite and reflects a post-magmatic mobility of uranium, which is greater under oxidizing conditions, causing a distinct higher U content. When comparing for the eU–K in Fig. 8b, a negligible correlation (R^2^ = 0.016) exists, indicating that potassium-bearing minerals such as K-feldspar and micas are not important controls on the distribution of uranium in the bostonite. Similarly, for the eTh–K plot (Fig. 8c), the R^2^ = 0.0016 also indicates that there is no meaningful relationship between thorium and potassium showing that thorium is incompatible with common K-bearing phenocrysts. The ratio plot of eTh/K with eTh/eU (Fig. 8d) shows an obvious pattern of uranium mobility within the samples. All but a few fall into the “Fixed-U” portion indicating that the uranium has generally stabilized; there are only a few samples that fall into the “Leached-U” region which implies uranium localized removal; this is likely due to hydrothermal alteration or oxidative weathering. The analyzed samples demonstrate patterns indicating secondary processes are responsible for controlling how radioelements distribute within the bostonite samples, as opposed to differentiation of magma at the time of initial formation. Uranium is observed to be the most mobile; thorium is observed at the least mobile level, while potassium interacts with both uranium and thorium geochemically to a limited extent.

### Radionuclide specific activity

The activity concentrations of ^238^U, ^232^Th, and ^40^K in the bostonite samples analyzed will be assessed by means of statistical analysis to determine the distribution, variability, and potential radiation hazards posed by these elements. Table 2 below shows the descriptive statistics for all 50 bostonite samples that were collected, including the mean, standard deviation, range, skewness, kurtosis, and coefficient of variation (CV%). The average concentration of ^238^U activity was 150 Bq kg⁻¹, and there is large variability in the concentrations measured (SD = 46 Bq kg^− 1^). These concentrations significantly exceed the value proposed by UNSCEAR (2010; 35 Bq kg^− 1^)^1^. Therefore, there was a great deal of geographic variation within the bostonite assemblage studied. All concentrations measured were in the range of 65–236 Bq kg^− 1^, demonstrating geographic variability and the possibility of varying types of uranium-bearing minerals. The coefficient of variance also indicates high variability in the distribution of uranium within the bostonite assemblage (CV = 31%), confirming our observations of the presence of accessory mineral phases (coffinite, uranophane and zircon) that are abundant in intrusive rocks of felsic to intermediate composition. The U-238 distribution shows that the data have a slight negative skewness (-0.12), meaning it has a fairly equal number of low and high ^238^U concentrations, but there is a slight tendency for lower values. The distribution also has a kurtosis of -1.10, indicating the distribution has less extreme values than a normal distribution (platykurtic); therefore, it exhibits a more uniform uranium enrichment with noted regions of increased ^238^U concentration, due to the presence of radioactive minerals such as coffinite, uranophane and zircon^17,18^.

In comparing thorium (^232^Th) with uranium, the distribution of activity concentrations of ^232^Th are more uniform than that of uranium, as evidenced by the relatively low coefficient of variance (CV) of ^232^Th of 16% and therefore, is also in keeping with its strong lithophile behaviour. During the evolution of a magma, Th is typically found in accessory minerals such as thorite, uranothorite, monazite and zircon^17,18^. The mean for ^232^Th activity concentrations are 103 Bq kg^− 1^ > of the worldwide value 45 Bq kg^− 1^^1^ and SD = 17 Bq kg^− 1^, respectively, while the Th activity concentrations range between 59 Bq kg^− 1^ and 140 Bq kg^− 1^, therefore, confirming that bostonite has a higher ^232^Th -enrichment than that of most crustal rocks. Skewness of (0.02) is nearly 0, and this supports the assumption of a very symmetrical distribution. Kurtosis value of (0.11) indicates that it is close to mesokurtic (normal). Therefore, it appears that thorium is well distributed throughout bostonite samples. This may be due to consistent mineralogy of the bostonite samples and/or no remobilization of thorium after magma intrusion. The mean values for the Th/U ratios also indicate that they fall within the expected range of intermediate to evolved igneous rock.

The mean radiometric potassium activity for ^40^K is reported as 1379 Bq kg^−1^ with a relatively high standard deviation (295 Bq kg^−1^). Activity concentrations ranged from 1033 to 2974 Bq kg^−1^; therefore, certain samples had significant enrichment of the potassium isotope, which supports the fact that bostonites contain a substantial quantity of K‐bearing minerals (K‐feldspar and biotite) compared to the UNSCEAR^1^ reference concentration of 412 Bq kg^−1^, which is over three times less than the average for ^40^K from this study (1379 Bq kg^−1^). With a coefficient of variation (CV) of 21%, the results indicate moderate variability. This variability is most likely explained by the large differences in the quantity of K‐feldspar found in these bostonite samples. The distribution of ^40^K is strongly skewed to the right (i.e., has a positive skewness) and has an extremely high kurtosis. This indicates that it is a leptokurtic distribution with a substantial amount of data located near the average, as well as a number of higher than average high-K samples or outliers. The outliers represent areas where high concentrations of ^40^K are likely the result of either sanidine-bearing or potassic alteration occurring within the sample area, and thus, may be indications of significant ^40^K enrichment in those areas.

**Table 2:** Descriptive statistics of bostonite samples studied at El Sela-Qash Amir area.

	*N*	Mean	SD	Min	Max	Skewness	Kurtosis	CV, %
^238^U, Bq kg^−1^	50	150	46	65	236	− 0.12	− 1.10	31
^232^Th, Bq kg^− 1^	50	103	17	59	140	0.02	0.11	16
^40^K, Bq kg^− 1^	50	1379	295	1033	2974	3.35	17.00	21

**Fig. 9:**
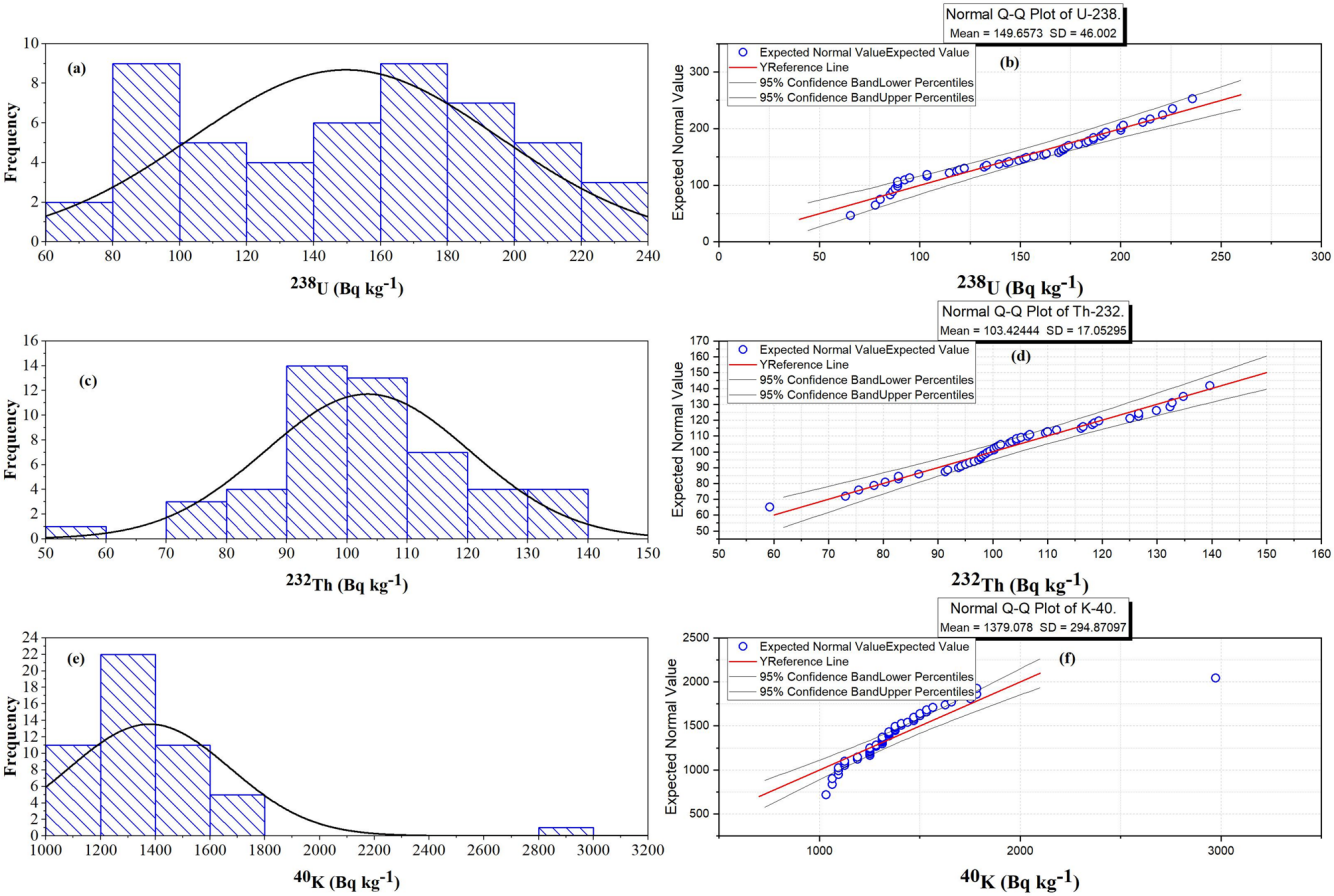
Frequency distribution and Q–Q plot of (**a**) ^238^U, (**b**) ^232^Th, and (**c**) ^40^K activity concentrations in bostonite at El Sela-Qash Amir area.

The frequency distribution in Fig. 9a,c,e and Q–Q plot in Fig. 9b,d,f display the distributions of ^238^U, ^232^Th and ^40^K in the bostonite sample to illustrate how these radionuclides are distributed across a range of concentrations. The distributions of ^238^U and ^232^Th show an approximate symmetry; this can also be seen in the bell-shaped histogram distributions for both radionuclides, while the majority of the Q-Q graph points are relatively close to the line of reference.

Data analysis and histogram construction were carried out using a multivariate statistical approach. IBM-SPSS version 21.0, a commercial statistics program, was used for these tasks. The distinct distributions of measured radionuclides in the bostonite samples were investigated using the Shapiro-Wilk Normality Test (Table 3). However, a histogram in Fig. 9a,b depicting the presence of ^238^U might imply grouping patterns due to the selection of bins. However, using statistical Shapiro-Wilk Normality Test (Table 3) has shown that the distribution of ^238^U can be regarded as approximately normal based on the results obtained from the Shapiro–Wilk test; W = 0.96 and *p* = 0.06 (*p* > 0.05) demonstrate no significant deviation from a normal distribution at the 0.05 significance level. The obtained values of skewness (− 0.12) and kurtosis (− 1.10) correspond to acceptable limits when examined in relation to the standard reference normal distribution curves. The Q–Q plot also indicates a similar pattern to that of a normal distribution; in fact, most of the sample observations are located very close to the theoretical Q–Q lines. The Shapiro–Wilk test also shows that ^232^Th behaves in a similar manner as it passes this test for normality (W = 0.98 and *p* = 0.42); while the distribution of ^40^K does not pass the test to indicate that it is normally distributed, where ^40^K is significantly different from normal distribution *p* < 0.001. This is consistent with this radioisotope having high skewness and localized enrichment.

**Table 3:** Results of Shapiro–Wilk normality tests.

	DF	Statistic	*p*-value
^238^U	50	0.96	0.06
^232^Th	50	0.98	0.42
^40^K	50	0.71	1.55E−08

The activity concentrations of ^238^U, ^232^Th, and ^40^K in bostonite samples are compared to previous investigations (Table 4) to determine their mean values. This comparison shows that the geological characteristics of the analyzed sites affect the activity concentrations of these elements.

**Table 4:** Comparison of ^238^U, ^232^Th and ^40^K activity concentration in the bostonite samples at El Sela-Qash Amir area with numerous world studies.

Country	^238^U		^232^Th		^40^K		References	
Egypt	150		103		1379		Present study	
Egypt	34		14		552		^55^	
Iraq	84.06		37.35		528.71		^56^	
Benin	44		129		1329		^57^	
Nigeria	30.4		3.31		222.25		^58^	
Turkey	0.7–186		0.5–249		1935		^59^	
India	18.2–30.3		47.9–62.2		176.6–248.1		^60^	
Spain	84		42		1138		^61^	

### Radiological risk assessment

The assessment of radiological hazards using bostonite samples includes Ra_eq_, H_ex_, H_in_, Iγ, D_air_, AED_out_, AED_in_, AGDE, and ELCR (see Table 5). There are moderate to high levels of radioactivity for all bostonite samples in this assessment. The Ra_eq_ values of all the bostonite samples range from a low of 309 Bq kg^− 1^ to a high of 559 Bq kg^− 1^, with an average Ra_eq_ value of 404 Bq kg^− 1^. Most bostonite samples exceed the recommended maximum limit of 370 Bq kg^− 1^^62^ for all construction grade material samples, indicating that many bostonite specimens contain a high proportion of uranium (^238^U), thorium (^232^Th), and potassium isotopes (^40^K).

**Table 5:** Radium equivalent activity (Ra_eq_ ), absorbed dose rate (D_air_ ), indoor, external indices annual outdoor effective dose (AED_out_ ), annual indoor effective dose (AED_in_), annual gonadal dose equivalent (AGDE) and excess lifetime cancer (ELCR) in the bostonite samples at El Sela-Qash Amir area.

Samples	Ra_eq_ (Bq/kg)	H_in_	H_ex_	Iγ	D_air_ (nG/h)	AED_out_ (mSv)	AED_in_ (mSv)	AGDE (mSv)	ELCR
S1	474	1.88	1.28	1.74	224	0.27	1.10	1.58	0.00096
S2	427	1.51	1.15	1.56	197	0.24	0.97	1.39	0.00085
S3	455	1.72	1.23	1.67	214	0.26	1.05	1.52	0.00092
S4	437	1.72	1.18	1.59	206	0.25	1.01	1.45	0.00088
S5	437	1.60	1.18	1.60	203	0.25	1.00	1.43	0.00087
S6	430	1.68	1.16	1.55	200	0.25	0.98	1.40	0.00086
S7	395	1.50	1.07	1.44	184	0.23	0.90	1.29	0.00079
S8	327	1.20	0.88	1.20	153	0.19	0.75	1.09	0.00066
S9	456	1.74	1.23	1.67	214	0.26	1.05	1.51	0.00092
S10	393	1.47	1.06	1.45	185	0.23	0.91	1.31	0.00079
S11	410	1.62	1.11	1.47	189	0.23	0.93	1.32	0.00081
S12	414	1.48	1.12	1.51	191	0.23	0.94	1.34	0.00082
S13	323	1.18	0.87	1.20	152	0.19	0.75	1.08	0.00065
S14	436	1.59	1.18	1.58	201	0.25	0.99	1.42	0.00086
S15	435	1.79	1.18	1.57	204	0.25	1.00	1.43	0.00088
S16	422	1.53	1.14	1.53	195	0.24	0.95	1.37	0.00084
S17	323	1.28	0.87	1.18	152	0.19	0.75	1.07	0.00065
S18	425	1.47	1.15	1.56	197	0.24	0.97	1.40	0.00085
S19	408	1.59	1.10	1.47	189	0.23	0.93	1.32	0.00081
S20	370	1.33	1.00	1.34	170	0.21	0.84	1.20	0.00073
S21	399	1.52	1.08	1.45	186	0.23	0.91	1.30	0.0008
S22	361	1.21	0.98	1.34	168	0.21	0.82	1.19	0.00072
S23	386	1.42	1.04	1.42	180	0.22	0.88	1.27	0.00077
S24	361	1.20	0.97	1.34	167	0.21	0.82	1.19	0.00072
S25	333	1.14	0.90	1.22	154	0.19	0.75	1.09	0.00066
S26	309	1.01	0.84	1.14	143	0.17	0.70	1.01	0.00061
S27	383	1.27	1.03	1.44	180	0.22	0.89	1.29	0.00077
S28	341	1.20	0.92	1.24	157	0.19	0.77	1.11	0.00068
S29	358	1.20	0.97	1.34	168	0.21	0.82	1.20	0.00072
S30	331	1.13	0.89	1.22	153	0.19	0.75	1.09	0.00066
S31	452	1.86	1.22	1.61	209	0.26	1.03	1.46	0.0009
S32	326	1.09	0.88	1.21	152	0.19	0.74	1.08	0.00065
S33	347	1.19	0.94	1.28	162	0.20	0.79	1.15	0.00069
S34	351	1.20	0.95	1.31	164	0.20	0.81	1.17	0.00071
S35	430	1.66	1.16	1.56	200	0.25	0.98	1.40	0.00086
S36	559	2.01	1.51	2.11	269	0.33	1.32	1.93	0.00115
S37	329	1.10	0.89	1.23	154	0.19	0.76	1.10	0.00066
S38	444	1.59	1.20	1.61	204	0.25	1.00	1.44	0.00088
S39	456	1.80	1.23	1.65	213	0.26	1.04	1.49	0.00091
S40	464	1.79	1.25	1.69	218	0.27	1.07	1.54	0.00094
S41	437	1.70	1.18	1.58	203	0.25	1.00	1.43	0.00087
S42	409	1.39	1.11	1.51	190	0.23	0.93	1.35	0.00081
S43	437	1.76	1.18	1.58	204	0.25	1.00	1.43	0.00088
S44	428	1.63	1.16	1.56	200	0.25	0.98	1.41	0.00086
S45	401	1.55	1.08	1.46	188	0.23	0.92	1.32	0.00081
S46	450	1.68	1.22	1.63	208	0.26	1.02	1.46	0.00089
S47	423	1.68	1.14	1.52	196	0.24	0.96	1.37	0.00084
S48	448	1.67	1.21	1.63	208	0.26	1.02	1.46	0.00089
S49	421	1.60	1.14	1.54	198	0.24	0.97	1.40	0.00085
S50	414	1.58	1.12	1.49	192	0.24	0.94	1.34	0.00082
Mean	404	1.49	1.09	1.48	188	0.23	0.92	1.33	0.00081
SD	51	0.25	0.14	0.18	24	0.03	0.12	0.17	0.0001
Min	309	1.01	0.84	1.14	143	0.17	0.70	1.01	0.00061
Max	559	2.01	1.51	2.11	269	0.33	1.32	1.93	0.00115

**Fig. 10:**
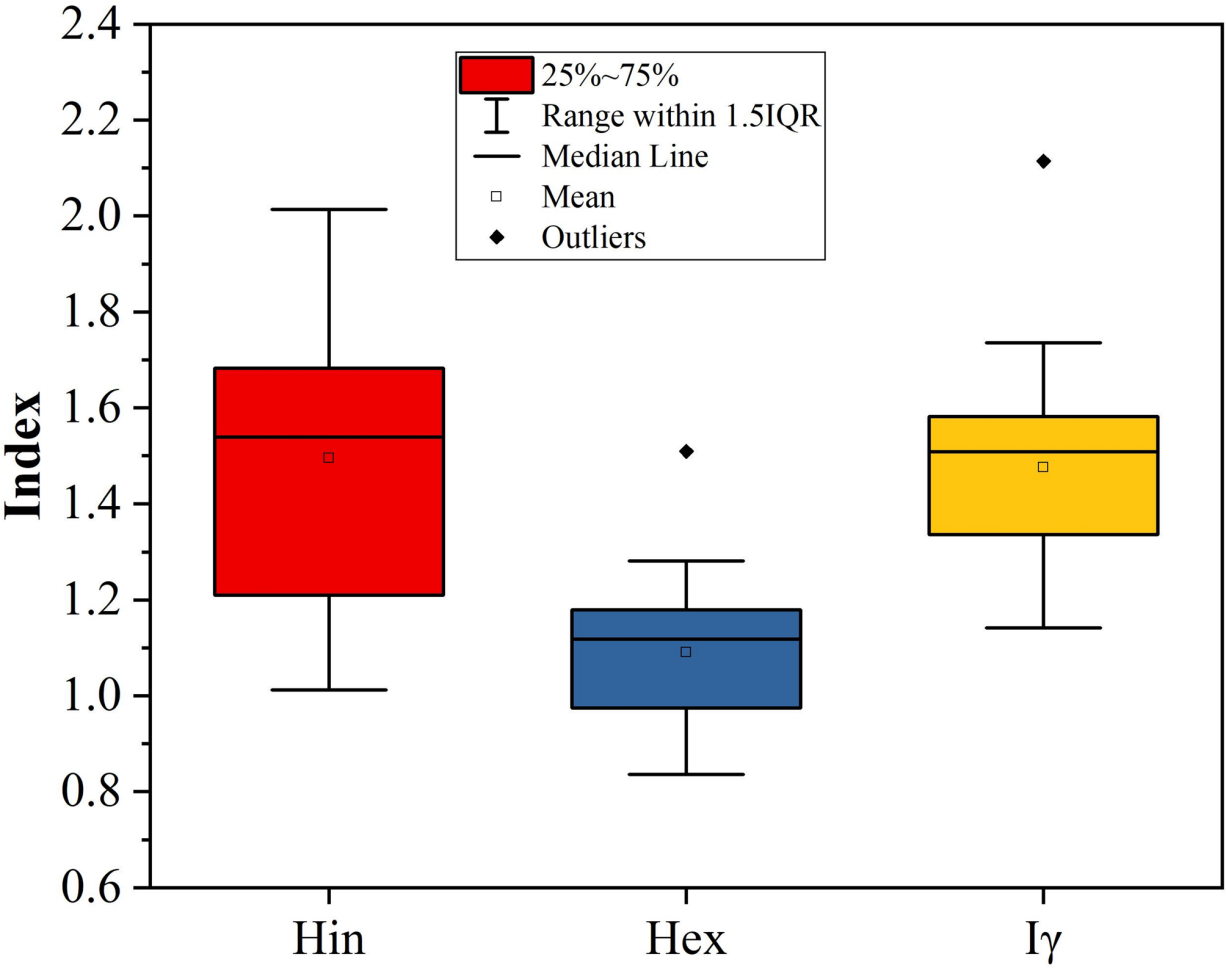
Hazard indices of bostonite samples at El Sela-Qash Amir area.

Both the H_ex_ and H_in_ mean approximately the same thing (1.09 for H_ex_ and 1.49 for H_in_) and have a very similar pattern. In most samples in Fig. 10, the mean values are greater than one^63^, which indicates that there is potential for uranium and/or its daughter products to be present in greater concentration if the rock were used indoors, which would lead to increased risk of radiation exposure from radon gas and/or its associated daughter products. When assessing the gamma index (I) of the H_ex_ and H_in_ samples, the ranges of the sample I values were 1.14 to 2.11, with an average of 1.48; the average Iγ value in Fig. 10 is greater than the maximum recommended limit (Iγ = 1^63^), for indoor construction use. This indicates that the samples assessed may be capable of generating elevated levels of gamma radiation dose rates.

This trend has also been confirmed from our calculation of absorbed dose rates in air (D_air_) in Table 5, which ranges from 143 to 269 nGy/h with an average of 188 nGy/h, approximately three times greater than the average value of 59 nGy/h (globally) for terrestrial gamma radiation^48^. The calculated annual effective doses (AED_in_ ranges from 1.01 to 1.93 mSv for indoor exposure (AED_out_ ranges from 0.17 to 0.33 mSv for outdoor exposure) exceed the limits of what is defined as normal background exposure internationally (0.07 mSv and 0.41 mSv for outdoor and indoor, respectively^48^), , suggesting that the principal contribution is due to the concentration of radioelement-bearing minerals in the bostonite. The calculated AGDE also varies considerably (1.01–1.93 mSv), corroborating the biological significance of our radiation measurements.

According to estimates, the Excess Lifetime Cancer Risk (ELCR) for bostonite samples is roughly in the range from 6.1 × 10^− 4^ to 1.15 × 10^− 3^ with an average of 8.1 × 10^− 3^. This ELCR is considered a low to moderate amount of increased cancer risk due to exposure to ionizing radiation from bostonite samples and is comparable to other igneous rock types that are commonly found throughout the United States. Many bostonite samples do exceed the national regulations on maximum allowable exposure to ionizing radiation (0.29–1.7 × 10^− 3^)^49^, and therefore, care should be used when evaluating potential uses for these rocks. In addition, the elevated levels of uranium, thorium, and potassium that are common in bostonite samples indicate that they have the potential for continued enhancement of their radioactivity and could exceed the limits as established by the national regulations.

The health implications are associated with the bostonite are may be due to uranium-bearing minerals, such as uranophane, represent a potential radiation hazard as they can release radioactive materials into the environment and a potential chemical hazard as they contain uranium as an extremely metallic elements such as Cu and Ni. It can release radioactive materials into the environment, therefore having a negative impact on human health through how easily they can enter into the human body from these minerals. What influences how quickly uranium enters into the human body from these minerals is its solubility and speciation (i.e., uranyl ion). The results of dissolution and inhalation (toxicological) tests on uranophane has shown that under both oxidizing conditions of stable oxidation state, uranophane can release U in a soluble (U(VI)) form, thus improving the potential chemical mobility of U through inhalation of dust or ingestion of altered rock. Recent work on uranophane has indicated that the hazardous impact of uranium-bearing dust on human health is determined by both the mineral type from which the uranium was released and the dissolution rate of such mineral, rather than by the bulk concentration of uranium (U)^64^. Therefore, the identification of uranophane within El Sela-Qash Amir bostonite provides additional support for the assertion that both radiological and chemical hazards exist from these materials, hence we recommend limiting the use of these materials in the construction of buildings applications.

### Statistical approach

There are significant relationships between the three radionuclides measured in this work, as well as between their respective risk indices (Table 6). ^238^U was found to have the greatest effect on radiological parameters (i.e., high positive correlation with the radon risk index (H_in_) of *r* = 0.94), strongly correlates with the other indices: Ra_eq_, H_ex_, Iγ, D_air_, AED_out_, AED_in_, AGDE and ELCR (all with r between 0.75 and 0.78), indicating that the variation of ^238^U concentration accounts for the majority of radiological risk in the bostonite samples studied. 232Th in comparison had only weak to negligible correlations with all of the hazard indices (*r* = -0.13 to 0.13), indicating that it does not contribute significantly to total exposure. Moderate to strong correlations (*r* = 0.54–0.63) exist between most of the hazard indices studied and ^40^K; these relationships indicate that ^40^K has a moderate to strong positive impact on external dose rates (and the associated exposure measures). The inter-radionuclide correlation examples depicted above show the negative correlation between ^238^U and K-40 (*r* = 0.13), ^238^U and ^232^Th (*r* = -0.38) and lack of correlation between ^232^Th and ^40^K (*r* = -0.04). The various relationships discussed above indicate that ^238^U represents the greatest contributor to radiological hazards within the bostonite sample’s distribution, followed by ^40^K, and then ^232^Th.

**Table 6:** Pearson correlation between natural radionuclides and the radiological hazard coefficients of bostonite, El Sela-Qash Amir area, Egypt.

	^238^U	^232^Th	^40^K	Ra_eq_	H_in_	H_ex_	Iγ	D_air_	AED_out_	AED_in_	AGDE	ELCR
^238^U	1.00	− 0.38	0.13	0.78	0.94	0.78	0.73	0.78	0.78	0.78	0.75	0.78
^232^Th	//	1.00	− 0.04	0.12	− 0.13	0.12	0.13	0.07	0.07	0.07	0.08	0.07
^40^K	//	//	1.00	0.54	0.37	0.54	0.62	0.59	0.59	0.59	0.63	0.59

**Fig. 11:**
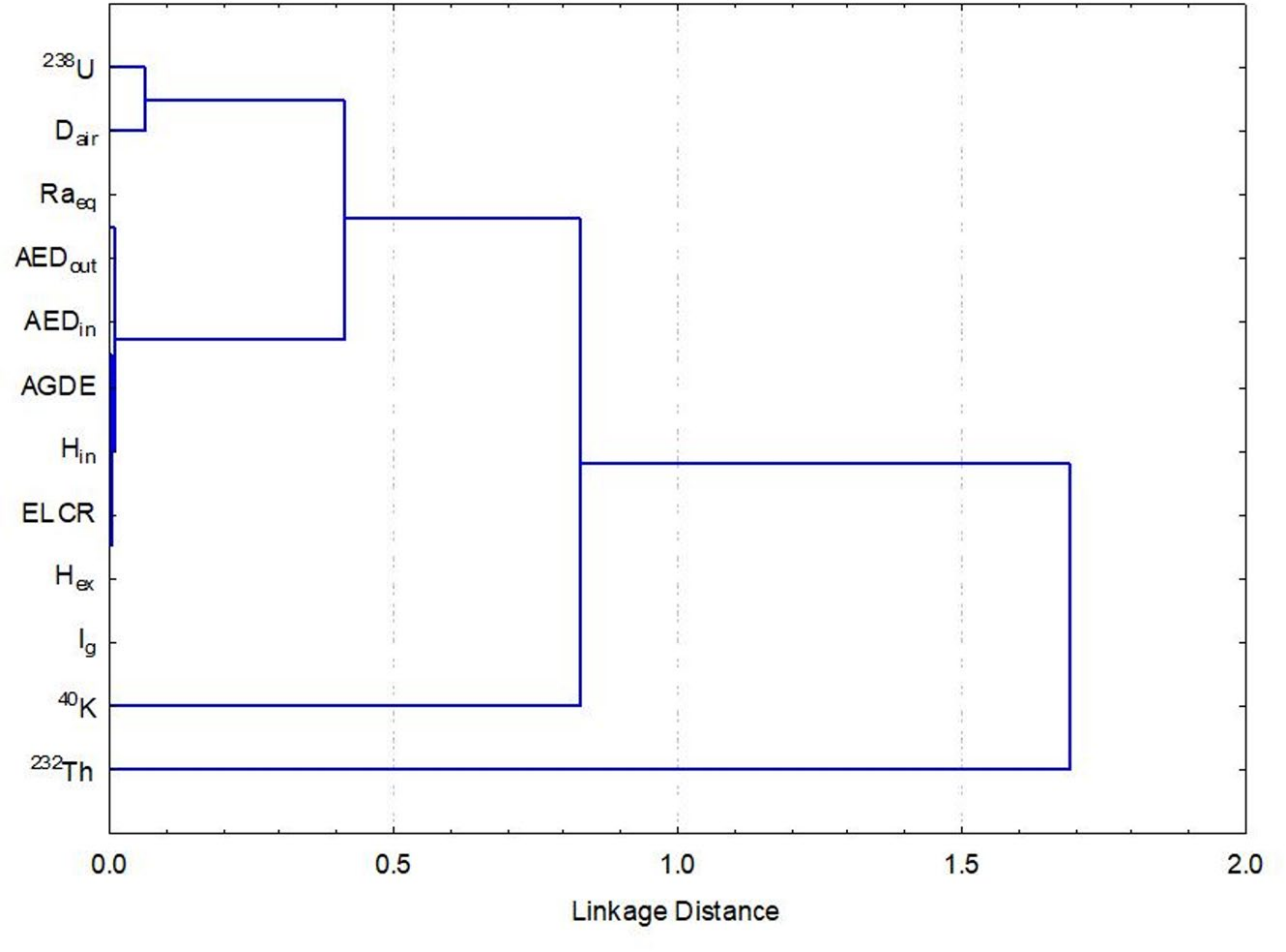
The clustering analysis of the radiological parameters in bostonite at El Sela-Qash Amir area.

Figure 11 illustrates the relationships between radioelement activity concentrations and radiological indices derived from hierarchy clustering analysis (HCA). ^238^U, ^232^Th, and ^40^K form separate branches indicating that these radionuclides have different origins based on their mineralogical host materials and geochemical behaviour within the bostonite. D_air_, Ra_eq_, AED_out_, AED_in_, AGDE, H_ex_, H_in_, and Iγ all cluster closely together with ^238^U and ^232^Th. Therefore, these two radionuclides are major contributors to gamma dose rate and risk index values. ^40^K appears in a separate branch due to its different statistical characteristics relative to U and Th, leading to their being less influential compared to ^238^U and ^232^Th on radiological hazard parameters. The presence of the different branches in HCA indicates that ^238^U and ^232^Th represent the dominant source of radiological hazards associated with the bostonite studied while K-40 provides only a secondary contribution.

**Fig. 12:**
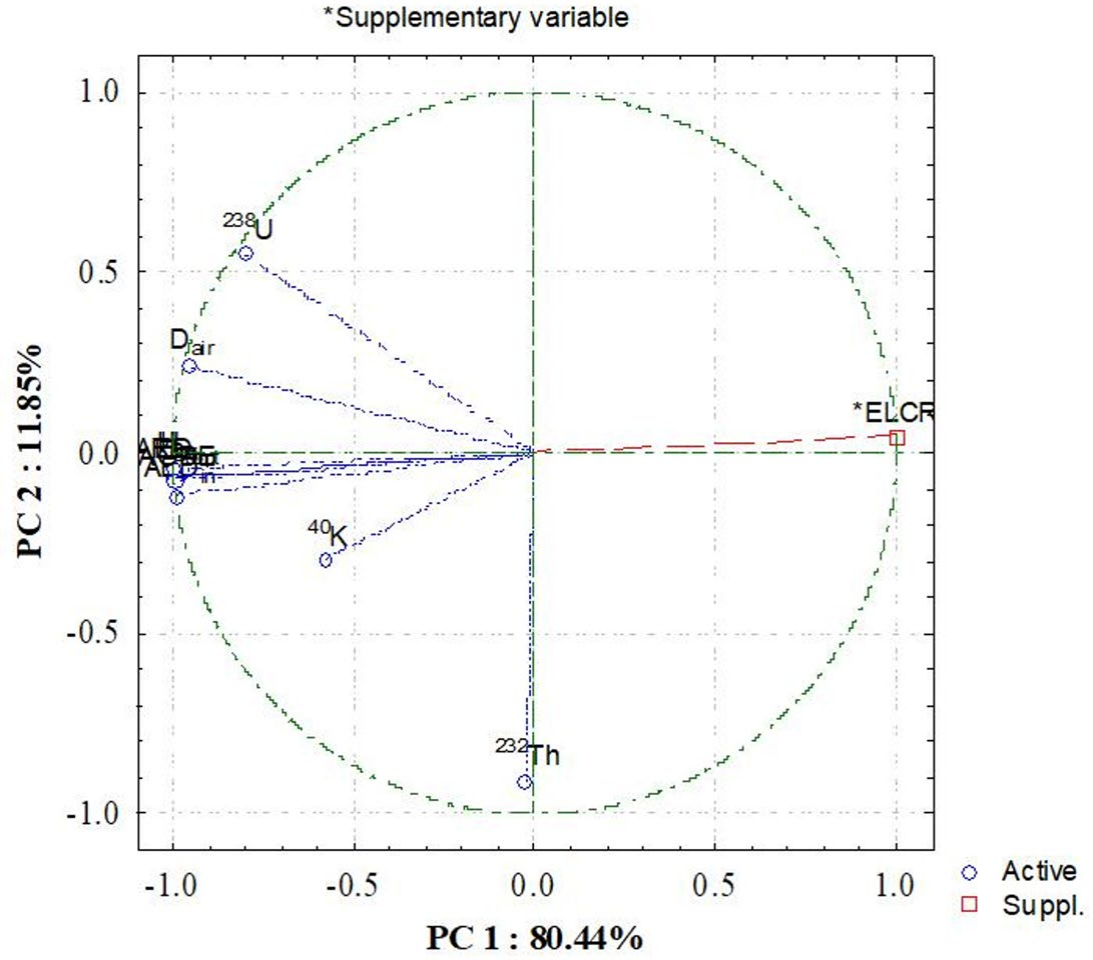
Principal component analysis (PC1 and PC2) for radiological data of bostonite at El Sela-Qash Amir area.

PCA analysis in Fig. 12 confirms that most of the radioactivity present at each sampling site is associated with ^238^U and ^232^Th. PC1 accounted for 80.44% of total variance and was heavily weighted with Ra_eq_, H_ex_, H_in_, Iγ, AGDE and dose parameters; they all point in the same direction as ^238^U and ^232^Th. PC2 (11.85% of variance) shows the differences in the trends of ^40^K over time; it is plotted orthogonally to ^238^U and ^232^Th, suggesting that it was derived from a very different geochemical setting when it was created and that its distribution is not similar to those elements. The ELCR index correlated highly with PC1, indicating that lifetime cancer risk is determined primarily by the joint effects of U and Th, given their substantially higher dose conversion factors and relatively even distribution across the samples collected. Therefore, the three combined methods of statistics, clustering and PCA demonstrate that while ^40^K may contain the highest levels of activity at individual sampling locations, the overwhelming majority of radiological risks are caused by ^238^U and ^232^Th as they correlate better with absorbed dose, radium equivalent activity, and hazard indices. Accordingly, the combined multivariate and correlation results provide a consistent interpretation of U (coffinite, uranophane and monazite as well as zircon)- and K (sanidine-bearing potassium) being the primary contributors toward raised Ra_eq_, D_air_, AED, hazard indices and ELCR values, which support the direct link between mineralogy/radiological hazard through these rocks.

## Conclusion

The geochemistry of El Sela bostonite displays a remarkable unique geochemical traits, distinguished by remarkable enriched alkalis, whereas Na_2_O and K_2_O reached up to 5.79 and 6.71 wt% with marked higher concentration of Fe_2_O_3_ that reached 5.52 wt%. The radioelements concentration reveal that Th and U reached up 25.44 and 17.21 mg kg^− 1^, whereas he Th/U ratio reached up 2.48 indicated positive correlation between them during magmatic fractionation. The bostonite shows anomalously high levels of U isotopes compared to the area where it originated, with U mobility and other post-magmatic processes needing evaluation as individual cases prior to any potential construction applications. The activity level for ^238^U (150 Bq kg^− 1^), ^232^Th (103 Bq kg^− 1^) and ^40^K (1379 Bq kg^− 1^) from the bostonite is much greater than the averages of U, Th and K from international radiological databases provided by UNSCEAR of 35, 45 and 412 Bq/kg, respectively. Although the amount of uranium in some of the samples exhibited moderate variability (CV = 31%) and oxide alteration due to radiological alteration, thorium remains relatively stable in all bostonite samples (CV = 16%) and has a nearly normally distributed concentration while there are cases of marked enrichment in potassium (CV = 21%), and non-normality due to the potassic alteration. Based on estimates of the hazard parameters, much bostonite samples contains multiple samples that come close to or exceed the recommended limits of safety, as evidenced by radium equivalent activity (Ra_eq_) of several samples near or just above the safety limit of 370 Bq kg^− 1^, and respective H_ex_ and H_in_ indices approaching 1.0 in places, suggesting a potential radiological concern upon use. Estimates of annual effective dose also revealed an unusually high level with one or more cases having indoor doses in excess of the 1 mSv y^− 1^ applicable limit. The results of this study indicate that while the bostonite from El Sela-Qash Amir has a desirable mineral such as (coffinite, uranophane, monazite and zircon) and geochemical profile, its radiological properties are significantly limiting the use for construction materials unless they are carefully selected, mixed or processed in such a way that they comply with international radiation safety regulations.

### Recommendation

Our recommendation to protect quarry employees, stone workers and transport personnel would be to develop regular radiological monitoring programs, occupational exposure assessments, and dust control programs, which will create lower inhalation risks to employees, as well as mitigate the presence of uranophane and other radioactive minerals. Furthermore, we suggest that ongoing programs include periodic GIS mapping to identify radiologically significant areas, also referred to as hot spots, located throughout the El Sela shear zone, and will aid in developing an overall strategy for the long-term management of environmental risk.

## Data Availability

The datasets used and/or analysed during the current study available from the corresponding author on reasonable request.
